# Does 20 Hz Transcranial Alternating Current Stimulation over the Human Primary Motor Cortex Modulate Beta Rebound Following Voluntary Movement?

**DOI:** 10.3390/brainsci14010074

**Published:** 2024-01-11

**Authors:** Mayu Akaiwa, Yuya Matsuda, Ryo Kurokawa, Yasushi Sugawara, Rin Kosuge, Hidekazu Saito, Eriko Shibata, Takeshi Sasaki, Kazuhiro Sugawara, Naoki Kozuka

**Affiliations:** 1 Graduate School of Health Sciences, Sapporo Medical University, Sapporo 060-8556, Japan; 2 Department of Occupational Therapy, School of Health Sciences, Sapporo Medical University, Sapporo 060-8556, Japan; 3 Major of Physical Therapy, Department of Rehabilitation, Faculty of Healthcare and Science, Hokkaido Bunkyo University, Eniwa 061-1449, Japan; shiba-e@do-bunkyodai.ac.jp; 4 Department of Physical Therapy, School of Health Sciences, Sapporo Medical University, Sapporo 060-8556, Japan; t-sasaki@sapmed.ac.jp (T.S.); kaz.sugawara@sapmed.ac.jp (K.S.); kozuka@sapmed.ac.jp (N.K.)

**Keywords:** transcranial alternating current stimulation, beta rebound, event-related synchronization, voluntary movement

## Abstract

Beta frequency oscillations originating from the primary motor cortex increase in amplitude following the initiation of voluntary movement, a process termed beta rebound. The strength of beta rebound has been reported to predict the recovery of motor function following stroke, suggesting therapeutic applications of beta rebound modulation. The present study examined the effect of 20 Hz transcranial alternating current stimulation (tACS) on the beta rebound induced by self-paced voluntary movement. Electroencephalograms (EEGs) and electromyograms (EMGs) were recorded from 16 healthy adults during voluntary movements performed before and after active or sham tACS. There was no significant change in average beta rebound after active tACS. However, the beta rebound amplitude was significantly enhanced in a subset of participants, and the magnitude of the increase across all participants was negatively correlated with the difference between individual peak beta frequency and tACS frequency. Thus, matching the stimulus frequency of tACS with individual beta frequency may facilitate therapeutic enhancement for motor rehabilitation.

## 1. Introduction

Transcranial alternating current stimulation (tACS) to the cerebral cortex can entrain endogenous neural oscillations [1,2,3], likely by promoting neuronal spike synchronization with the stimulation frequency [4]. For instance, tACS at alpha (8–12 Hz) frequency was found to enhance human alpha amplitude during resting state after 10 min of stimulation [1], while tACSs at alpha and beta frequencies (13–30 Hz) were reported to increase alpha and beta oscillations, respectively, but not other frequency bands [5]. tACS induces a frequency-dependent modulation of ongoing oscillatory activity, and the effective stimulation frequency is thought to match the targeted brain oscillations.

Modulations in beta oscillation are measured in the sensorimotor cortex and associated with motor control [6,7,8]; consistent with entrainment, tACS has been demonstrated to increase the amplitude of beta oscillations at resting state [5,9] and voluntary movement [10]. The size of motor-evoked potentials induced by transcranial magnetic stimulation over the motor cortex significantly increases during tACS at 20 Hz compared with other frequencies [11]; therefore, beta tACS was found to increase motor cortex excitability [11,12]. Furthermore, a previous study applied transcranial alternating current stimulation (tACS) at 20 Hz over the left primary motor cortex (M1) during a serial reaction time task, and tACS facilitated motor learning and stabilized newly learning motor sequences [13]. However, other studies have found reduced motor cortex excitability [14] or no change [15].

Most previous studies on beta tACS have examined effects on motor output, but beta oscillations also reflect motor cortex deactivation and afferent input. In the motor cortex, powerful suppression of the beta frequency, termed event-related desynchronization (ERD), occurs prior to movement onset and during movement execution. Following suppression, the beta rhythm increases above baseline, which is known as post-movement event-related synchronization (ERS), or beta rebound. It has been proposed that beta rebound reflects cortical deactivation (the so-called idling hypothesis) [16] and (or) sensory afferent processing [17]. The beta rebound has also been examined in various neurological disorders such as stroke, Parkinson’s disease, and amyotrophic lateral sclerosis [18,19,20,21], and reported to correlate with post-stroke motor function [22] as well as with the severity of corticospinal damage [21]. Therefore, the beta rebound after voluntary movement could serve as a valuable biomarker for neurological assessment. However, there are no reports of tACS effects on the beta rebound following voluntary movement. In the present study, we examined the effects of tACS over the M1, which may be a primary beta rebound generator [23], on beta rebound evoked by self-paced voluntary movements. Based on previous studies reporting that tACS can enhance beta rhythm [5,9] and beta ERD [10], we hypothesized that 20 Hz tACS over M1 would enhance beta rebound.

## 2. Materials and Methods

### 2.1. Participants

The minimum sample size was estimated from the partial η-squared using G*power 3.1.9.4, as described in a previous study [24]. Briefly, the effect size was set at 0.42, yielding a minimum sample size of 14. We recruited 16 right-handed healthy young adults (age [mean ± standard deviation] 22.81 ± 1.59 years; 9 men and 7 women) with no history of neurological, orthopedic, or psychiatric disorders; no chronic medication that could affect sensory processing or movement; or no metals located in the head. All participants provided written informed consent. The study was approved by the ethics committee of Sapporo Medical University (No. 3-1-12) and conformed to the tenets of the 1964 Helsinki Declaration and later amendments.

### 2.2. Experimental Procedure

**Figure 1:**
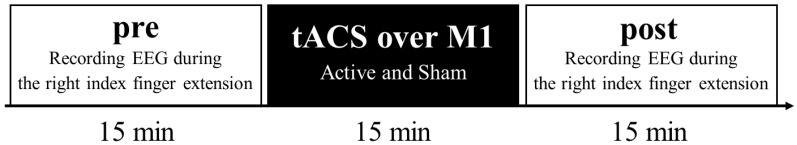
Time course of the alternating current stimulation (tACS) sessions and electroencephalogram (EEG) measurements. All participants received active tACS (active condition) and sham tACS (sham condition). tACS was applied over the left primary motor cortex at 20 Hz. EEG during the right index finger extension was recorded simultaneously before (pre) and after (post) tACS.

This study employed a cross-over design to investigate the effect of active versus sham tACS on beta rebound following voluntary movement. All participants received active tACS (active condition) and sham tACS (sham condition) at least 3 days apart with counterbalancing to prevent bias from carryover effects. During active or sham tACS, the participants were instructed to relax and look at a fixation cross displayed 1.5 m in front of them. Both the EEG and electromyogram (EMG) were recorded simultaneously before (pre) and after (post) tACS (Figure 1). For the voluntary movement component, participants were instructed to extend their right index finger 60 times at around 5 s intervals in one set. Participants performed three such sets (180 individual finger extensions) both pre and post tACS.

### 2.3. Transcranial Alternating Current Stimulation Protocol

tACS was applied using a DC Stimulation-Plus instrument (NeuroConn, Ilmenau, Germany) through a pair of 7 × 5 cm (35 cm^2^) rubber electrodes covered with saline-soaked sponges. The target electrode was placed at C3 according to the international 10–20 system and the reference electrode was placed above the contralateral orbit [10]. The intensity of stimulation was set to 1 mA peak-to-peak and the stimulation frequency was 20 Hz [10,25]. The stimulation duration was set to 15 min in the active condition and 30 s in the sham condition. To minimize skin sensations during stimulation, electrode impedance was maintained below 10 kΩ [26]. Each active stimulation session included 10 s ramp-up and ramp-down periods. After 1 min of tACS, participants scored subjective experiences of itching, tingling, and phosphenes on an 11-point numeric rating scale (NRS), with 0 indicating no sensation and 10 representing the strongest imaginable sensation.

### 2.4. Data Acquisition

All electrophysiological recordings were acquired using a Neurofax system (EEG-1200, Nihon Kohden, Tokyo, Japan). Electroencephalograms were recorded with Ag/AgCl disk electrodes placed on the scalp at F3, F4, C3, C4, P3, P4, Fz, Cz, and Pz, according to the International 10–20 system; 2 reference electrodes placed on the left and right earlobes (A1 and A2). The electrooculogram (EOG) was recorded from the right suborbital region and 2 electrodes were placed over the right extensor indicis proprius to measure the EMG. The impedance of all electrodes was maintained below 5 kΩ. EEG and EMG signals were bandpass-filtered at 0.08 to 300 Hz and digitized at a sampling rate of 1000 Hz.

### 2.5. Signal Processing

#### 2.5.1. Preprocessing

The EMG, EEG, and EOG records were analyzed using the Brainstorm toolbox running in MATLAB R2019a [27]. The EMG records were Notch-filtered at 50 Hz and rectified. Movement onset was defined when the rectified EMG voltage exceeded the baseline by mean ± 2 standard deviations (SDs) [28]. The EEG and EOG data were also Notch-filtered at 50 Hz and then segmented into epochs from 3000 ms prior to 2000 ms after movement onset. Epochs in which the EEG or EOG waveform exceeded 150 µV were rejected. In addition, EEG records with movement onset appearing less than 5 s after the last movement were rejected.

#### 2.5.2. Analysis of Beta Rebound

**Figure 2:**
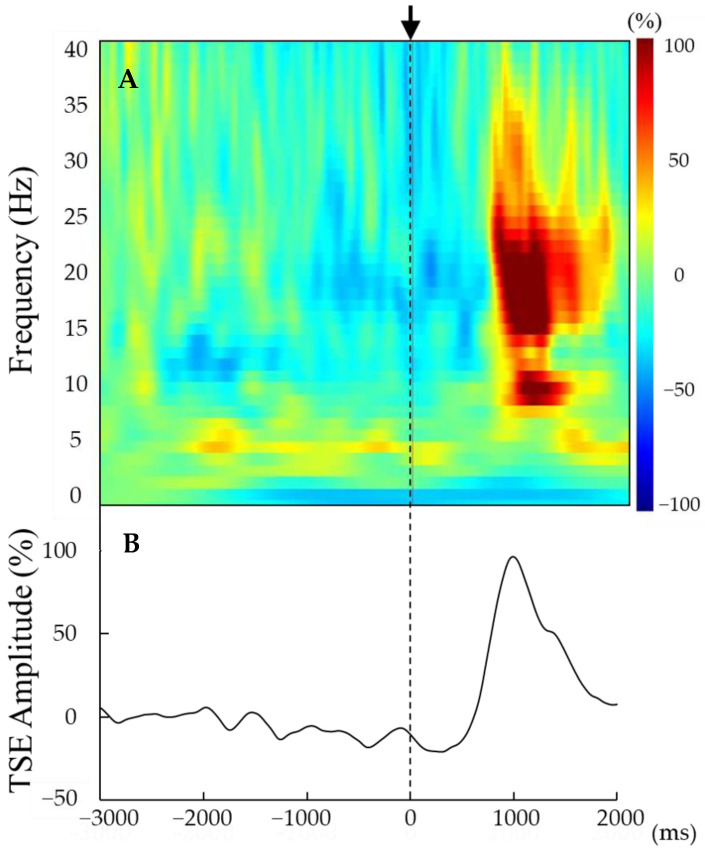
(**A**,**B**) Representative time–frequency analysis results (**A**) and TSE curves (**B**) derived from the averaged entire EEG epoch (from 3000 ms before to 2000 ms after movement onset) for a single participant. Movement onset (t = 0) is indicated by the vertical line. Beta rebound was observed approximately 1000 ms after movement onset (0 ms). The strength of beta rebound was detected by the peak amplitude of the TSE curve.

The beta rebound was recorded at electrode C3 placed over the contralateral sensorimotor cortex [17]. Time–frequency analysis was performed to visualize the peak frequency of the beta rebound (frequency of peak amplitude/power) under 4 conditions (Figure 2A): pre-active tACS, post-active tACS, pre-sham tACS, and post-sham tACS. For this purpose, we employed Morlet wavelet analysis as implemented in Brainstorm, based on a mother wavelet with a central frequency at 1 Hz and temporal resolution of 3 s full-width at half-maximum [29,30]. The baseline was determined as the average from 3000 to 2000 ms before finger movement.

The strength of the beta rebound was determined by temporal spectral evolution (TSE; Figure 2B) [31,32]. Previous studies have conducted TSE using the bandpass filter of a 10 Hz band (e.g., 15–25 Hz) [33]. However, our current study adopted a 3 Hz band during TSE due to certain participants detecting beta rebound within a limited frequency band. Briefly, the 3 Hz frequency band containing the peak beta rebound was extracted for each participant from within the 15–30 Hz band. Again, baseline was defined within 3000 to 2000 ms prior to movement onset, and the strength of beta rebound converted to a relative value according to the formula (x − μ)/μ × 100, where x is the peak voltage amplitude at each time point from 500 to 2000 ms after movement onset and μ is the mean baseline [33]. The median value of the 3 Hz band extracted for each participant was defined as the individual frequency at which the beta rebound was maximum.

### 2.6. Statistical Analysis

All statistical analyses were performed using IBM SPSS 24.0.0.0 software (IBM Corp., New York, NY, USA). Differences in TSE peak amplitude were tested by two-way repeated measures ANOVA with the main factors of CONDITION (active or sham tACS) and TIME (pre and post tACS). We investigated the effect of tACS on beta rebound when the individual maximum beta rebound frequency was close to the tACS stimulus frequency (20 Hz). The relationship between the change in strength of beta rebound following tACS (post tACS minus pre tACS) and the difference between individual peak frequencies at beta rebound and stimulus frequency (20 Hz) was examined using Spearman’s correlation analysis. Differences in NRS values for sensory experience (itching, tingling, and phosphenes) between active and sham conditions were evaluated using Wilcoxon’s signed-rank test.

## 3. Results

### 3.1. Individual Variation in Beta Rebound Modulation by tACS

**Figure 3:**
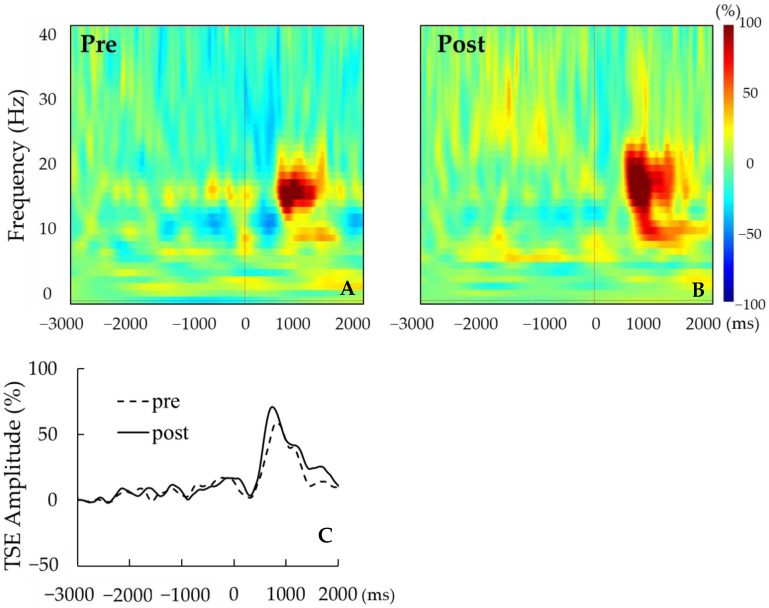
(**A**,**B**) Representative time–frequency analyses pre tACS (**A**) and post tACS (**B**) for a single participant. (**C**) Representative TSE curves pre tACS (dotted line) and post tACS (solid line). For this participant, the strength of beta rebound was greater post tACS.

**Figure 4:**
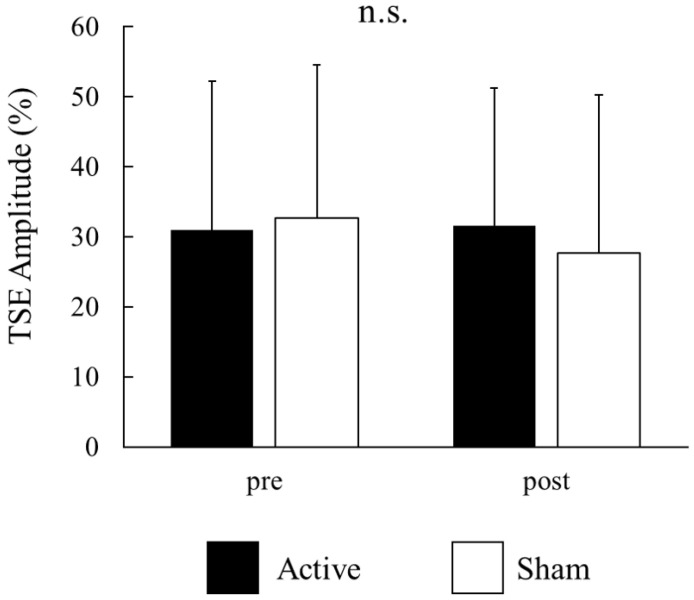
Effects of active and sham tACS on beta rebound strength for all participants. Values are presented as the mean ± standard deviation. There were no significant main effects of CONDITION (active vs. sham) and TIME (pre vs. post tACS), and no significant CONDITION × TIME interaction. There were no significances (n.s.).

For each participant, the signal-averaged EEG in the pre tACS of the active condition was obtained from 130 ± 22 records (mean ± S.D); that in the post tACS of the active condition was obtained from 134 ± 25 records; that in the pre tACS of the sham condition was obtained from 129 ± 25 records; and that in the post tACS of the sham condition was obtained from 131 ± 34 records. A beta rebound after movement onset was detected in 14 of the 16 participants. Figure 3A,B show the time–frequency analyses of EEG records obtained before and after tACS, while Figure 3C shows the TSE curves of pre tACS and post tACS. Beta rebound strength values and peak latencies of TSE curves are presented in Table 1 and Table 2, respectively. Two-way repeated measures ANOVA for the strength of the beta rebound indicated no significant main effects of CONDITION (F_(1,13)_ = 0.26, *p* = 0.62) and TIME (F_(1,13)_ = 2.33, *p* = 0.15), and no CONDITION × TIME interaction (F_(1,13)_ = 2.72, *p* = 0.12; Figure 4). At the individual level, however, 7 out of 14 participants with a measurable beta rebound demonstrated an increased strength of beta rebound after active tACS, while the remaining participants exhibited a decrease or no change.

### 3.2. Beta Rebound Enhancement by tACS near the Individual Peak Frequency

**Figure 5:**
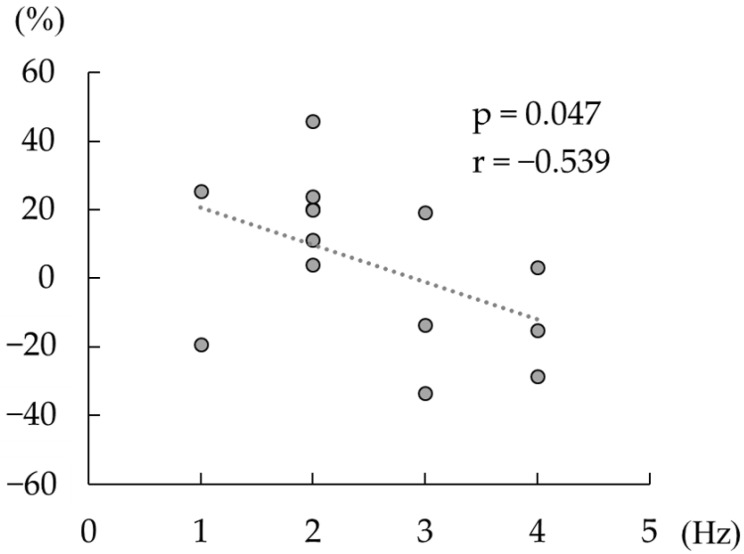
Correlation between the change in the strength of beta rebound and the difference between the individual beta rebound peak frequency and the tACS frequency (20 Hz). The dashed line shows a regression line. The horizontal axis shows the absolute value of the difference between the tACS frequency and the frequency at which the individual beta rebound strength was maximal. The vertical axis shows the change in beta rebound strength between the pre-tACS and post-tACS conditions (>0 defined as increased the strength of beta rebound post tACS versus pre tACS).

Spearman’s correlation analyses revealed a significant negative correlation between the change in the beta rebound strength after active tACS, and the difference between the individual peak frequency at beta rebound and tACS frequency (*p* < 0.05, ρ = −0.54; Figure 5), whereas no such correlation was detected following sham tACS (*p* = 0.77, ρ = −0.09).

### 3.3. Subjective Sensations during tACS

There were significant differences in NRS values for tingling (*p* = 0.03) and phosphenes (*p* < 0.01) following active tACS compared with sham tACS, but no difference in itching score (*p* = 0.37).

## 4. Discussion

The present study found three important results. First, the majority of participants (14 out of 16) demonstrated a beta rebound over the contralateral sensorimotor cortex (C3) after movement onset. Second, although there was no average change in the strength of the beta rebound after active 20 Hz tACS for the entire cohort, seven participants did demonstrate enhanced strength. Third, the effect was greater when the individual peak frequency at beta rebound power was closer to the tACS stimulus frequency (20 Hz). Thus, tACS entrained to the endogenous beta frequency can effectively increase the strength of the beta rebound.

### 4.1. Matching of the tACS Frequency with Individual Peak Beta Rebound Frequencies Enhances the Beta Rebound Amplitude

The administration of 20 Hz tACS over M1 increased the amplitude of beta rebound in 7 out of 16 participants, and the effect was larger when the individual peak frequency at beta rebound was closer to 20 Hz (the tACS frequency). Previous studies have reported that alpha tACS closer to the individual participant’s intrinsic alpha frequency produced stronger entrainment [34,35]. Moreover, a recent study reported no significant effects of 10 Hz and 20 Hz tACS on local beta and alpha band frequencies, respectively [36], suggesting that a fixed stimulation frequency substantially different from the peak endogenous frequency is ineffective for modulation. Furthermore, a previous study reported that when integrated with neuroimaging, simulations of electric fields can be used to derive qualitative measures of the spatial correlation between the electric field and target and the mismatch between stimulation frequency and frequency of the target oscillation [37]. These measures explained a substantial proportion of variance (51–87%) of the tACS effect on increasing power. Thus, it was reported that the separation between stimulation and peak endogenous frequency accounts for the variance in the strength of the tACS-induced aftereffect [37]. Recent studies have also reported that a tACS frequency matched to an individual frequency has stronger effects on cortical oscillation and behavior [1,38,39,40]. Our findings indicate that this frequency matching can also enhance the strength of the beta rebound.

In our investigation, the results revealed the offline effect of tACS. tACS-induced offline effects can be explained through spike-timing-dependent plasticity (STDP) [1]. According to the STDP rule, synapses in circuits resonating at a frequency similar to repetitive inputs are strengthened during stimulation. Subsequent to stimulation, these synaptic changes persist and result in enhanced neural activity at the resonance frequencies of these circuits. A previous study documented the sustained increase in beta oscillations induced by beta tACS for a minimum of 60 min [41]. Given the similarity of our tACS protocol to that of the mentioned study (e.g., stimulus site and stimulus frequency), the effects of tACS in our investigation may exhibit stability. Recent research has proposed that the influence of tACS on event-related oscillatory activity is likely attributed to a more pronounced enhancement of oscillation power preceding the event [42]. Therefore, our results could demonstrate that the increase in beta rebound among these seven participants can be attributed to the enhancement of beta power observed at baseline.

### 4.2. Inter-Individual Variation in Beta Rebound Modulation by tACS among Participants

In this study, 2 participants exhibited no detectable beta rebound following self-paced voluntary movement; variability in the strength of beta rebound was substantial among the other 14 participants. A highly variable beta rhythm was also reported during passive movement, with undetectable beta oscillations in some individuals [43]. It has been reported that the beta rebound is generated at M1 [23] and that beta rebound modulation should be measured by magnetoencephalogram (MEG) compared with EEG [44]. In general, EEG is a better detector of radial sources, while MEG is a more sensitive detector of tangential sources [45]. Thus, the beta rebound measured here likely reflects the activity of neurons positioned radially relative to the scalp surface, such as in the fissure of M1. The signal-to-noise ratio of a typical EEG is lower than that of MEG [44], and so may be less sensitive for the detection of a beta rebound. Therefore, in future studies, we will combine MEG and EEG to examine beta rebound modulation in all participants, possibly including those without detectable signals on the EEG.

### 4.3. Prospects for Stroke Rehabilitation

Neuronal oscillations within the beta frequency range (15–30 Hz) constitute the foundation of motor control and are associated with GABAergic activity in humans [46]. The beta rhythm might play an important role as a neurophysiological marker of motor system function and dysfunction. A previous study indicated minimal within-subject variability for both beta rhythm and beta rebound power [47], establishing their utility in fundamental research and clinical studies. The strength of beta rebound to impaired hand stimulation during the acute phase has been reported to correlate with the recovery of motor function at 1 and 12 months post stroke [18] and contributes to predicting motor performance in stroke patients [48]. Beta rebound has been considered to be useful in detecting alterations in cortical excitability, facilitating the monitoring of intervention effects across different recovery stages [18]. If tACS can enhance beta rebound evoked by voluntary movements, it may be applied to neurorehabilitation in stroke patients. The portability and simplicity of tACS make it a practical tool for bedside rehabilitation. However, previous studies investigating the effects of tACS on stroke patients have often used fixed frequencies, which may limit the individual efficacy [28,49]. Our results suggest that a tACS protocol with individualized frequency may be more effective in enhancing beta rebound for neurorehabilitation. In a future study, we will investigate the effect of tACS on beta rebound and motor function in stroke patients.

### 4.4. Limitations

A major limitation of this study is insufficient blinding between active and sham conditions, as evidenced by the significant difference in sensory NRS scores during tACS. A previous study similarly found that a parietal tACS montage induced phosphenes even at a weak stimulation intensity of 0.25 mA [50]. Moreover, 20 Hz tACS was reported to elicit skin sensations [51]. We used the M1 orbital tACS montage, whereas the M1 shoulder montage may minimize phosphene perception [52]. Furthermore, keeping electrode resistance as low as possible may reduce skin sensations. In future studies, it is necessary to fully blind the tACS condition by reducing skin sensation and phosphene perception.

## 5. Conclusions

We examined the effects of tACS over the primary motor cortex (M1) on beta rebound evoked by self-paced voluntary movements. The present study demonstrates that tACS over M1 can effectively modulate the strength of the beta rebound elicited by voluntary movement if the stimulation frequency is close to the peak frequency at beta rebound. We should combine EEG and MEG with individualized tACS frequencies to fully access the effects of beta rebound modulation. In a future study, we will investigate the effect of tACS on beta rebound and motor function in stroke patients.

## Figures and Tables

**Table 1:** Beta rebound strength before (pre) and after (post) active and sham tACS.

	Active		Sham	
	Pre	Post	Pre	Post
Mean, %	30.9	31.5	31.9	28.4
SD	21.3	21.8	19.0	23.0

**Table 2:** Peak latency of TSE curves before (pre) and after (post) active and sham tACS.

	Active		Sham	
	Pre	Post	Pre	Post
Mean, ms	1078	1043	1103	1078
SD	271	325	287	238

## Data Availability

The data are not publicly available due to ethical restrictions. The data presented in this study are available on request from the corresponding author.
